# Porphyran Attenuates Neuronal Loss in the Hippocampal CA1 Subregion Induced by Ischemia and Reperfusion in Gerbils by Inhibiting NLRP3 Inflammasome-Mediated Neuroinflammation

**DOI:** 10.3390/md22040170

**Published:** 2024-04-11

**Authors:** Dae Won Kim, Tae-Kyeong Lee, Ji Hyeon Ahn, Se-Ran Yang, Myoung Cheol Shin, Jun Hwi Cho, Moo-Ho Won, Il Jun Kang, Joon Ha Park

**Affiliations:** 1 Department of Biochemistry and Molecular Biology, Research Institute of Oral Sciences, College of Dentistry, Gangneung-Wonju National University, Gangneung 25457, Republic of Korea; kimdw@gwnu.ac.kr; 2 Department of Food Science and Nutrition, Hallym University, Chuncheon 24252, Republic of Korea; tk_lee@hallym.ac.kr; 3 Department of Physical Therapy, College of Health Science, Youngsan University, Yangsan 50510, Republic of Korea; jh-ahn@ysu.ac.kr; 4 Department of Cardiovascular Surgery, School of Medicine, Kangwon National University, Chuncheon 24341, Republic of Korea; seran@kangwon.ac.kr; 5 Department of Emergency Medicine, Kangwon National University Hospital, School of Medicine, Kangwon National University, Chuncheon 24289, Republic of Korea; dr10126@naver.com (M.C.S.); cjhemd@kangwon.ac.kr (J.H.C.); mhwon@kangwon.ac.kr (M.-H.W.); 6 Department of Anatomy, College of Korean Medicine, Dongguk University, 123 Dongdae-ro, Gyeongju 38066, Republic of Korea

**Keywords:** hippocampus, microglia, NLRP3 inflammasome complex, pro-inflammatory cytokines, pyramidal cells, sulfated polysaccharide

## Abstract

Porphyran, a sulfated polysaccharide found in various species of marine red algae, has been demonstrated to exhibit diverse bioactivities, including anti-inflammatory effects. However, the protective effects of porphyran against cerebral ischemia and reperfusion (IR) injury have not been investigated. The aim of this study was to examine the neuroprotective effects of porphyran against brain IR injury and its underlying mechanisms using a gerbil model of transient forebrain ischemia (IR in the forebrain), which results in pyramidal cell (principal neuron) loss in the cornu ammonis 1 (CA1) subregion of the hippocampus on day 4 after IR. Porphyran (25 and 50 mg/kg) was orally administered daily for one week prior to IR. Pretreatment with 50 mg/kg of porphyran, but not 25 mg/kg, significantly attenuated locomotor hyperactivity and protected pyramidal cells located in the CA1 area from IR injury. The pretreatment with 50 mg/kg of porphyran significantly suppressed the IR-induced activation and proliferation of microglia in the CA1 subregion. Additionally, the pretreatment significantly inhibited the overexpressions of nucleotide-binding domain, leucine-rich-containing family, pyrin domain-containing protein-3 (NLRP3) inflammasome complex, and pro-inflammatory cytokines (interleukin 1 beta and interleukin 18) induced by IR in the CA1 subregion. Overall, our findings suggest that porphyran exerts neuroprotective effects against brain IR injury, potentially by reducing the reaction (activation) and proliferation of microglia and reducing NLRP3 inflammasome-mediated neuroinflammation.

## 1. Introduction

Transient brain ischemia (brain IR) is a temporary interruption of blood flow to the entire brain, which occurs due to various factors, such as cardiac arrest, shock, and asphyxia [1,2]. This interruption causes a deficiency of oxygen and glucose, leading to neuronal damage or death (loss) [3]. The restoration of blood flow, known as reperfusion, is a critical step in the management of brain IR; however, it initiates additional detrimental processes, such as calcium overload, oxidative stress, and an inflammatory response, ultimately leading to severe brain damage [4,5,6]. The hippocampus is an important structure located in the forebrain and is one of the most vulnerable brain areas to cerebral IR injury [4]. In particular, among the hippocampal subregions, the CA1 subregion (area) is recognized to be particularly susceptible to IR injury, resulting in the selective and massive death (loss) of principal neurons, known as pyramidal cells, in the CA1 subregion several days after forebrain IR [7,8]. The underlying mechanisms contributing to IR-induced loss of the CA1 pyramidal cells are complex and have been widely studied [9,10]. At the same time, many researchers have discovered bioactive compounds displaying neuroprotection and investigated their potential mechanisms in animal models of transient cerebral ischemic insults.

Bioactive compounds derived from natural sources have garnered significant attention as potential candidates for preventing and/or treating brain disorders, including cerebral ischemic insults, due to their diverse biopharmacological activities and safety aspects [11,12]. In that connection, recent studies have proven that polysaccharides extracted from marine algae possess strong neuroprotective potential against cerebral IR injury. For instance, pretreatment with fucoidan significantly reduces cerebral infarction and neurological deficit induced by transient focal cerebral ischemia in rats [13] and attenuates the death of pyramidal cells in the hippocampal CA1 subregion after forebrain IR injury in gerbils [14]. Additionally, pretreated laminarin successfully protects hippocampal CA1 pyramidal cells against IR injury in the gerbil forebrain [15,16].

Porphyran is a sulfated polysaccharide found in various species of red algae, such as *Pyropia haitanensis* and *Pyropia yezoensis*. The molecular weight of porphyran can vary significantly due to various factors, such as the place of collection, time, and extraction methods. These variations may influence its bioavailability and biological activities [17]. It has been reported that porphyran displays diverse bioactivities, including anti-inflammatory and antioxidant effects in in vitro studies [17,18]. These bioactivities have led to the recent studies on porphyran in animal models of neurological disorders. For example, Zhang et al. (2020) demonstrated that porphyran offered protective effects against the neurotoxicity induced by amyloid β peptide in mice with Alzheimer’s disease [19]. Additionally, Liu et al. (2018) reported that oligo-porphyran, a degradation product of porphyran, protected dopaminergic neurons in mice with Parkinson’s disease induced by 1-methyl-4-phenyl-1,2,3,6-tetrahydropyridine [20]. However, based on our current knowledge, the neuroprotective potential of porphyran against IR injury in the forebrain has not been examined. Therefore, this experiment was conducted to examine the neuroprotective effects of porphyran and to explore its inhibition of neuroinflammation mediated by nucleotide-binding domain, leucine-rich-containing family, and pyrin domain-containing protein-3 (NLRP3) inflammasome—a multiprotein complex playing a pivotal role in regulating the innate immune system and inflammatory signaling [21]—in the hippocampal CA1 subregion using a gerbil model of forebrain IR injury, which is commonly used to evaluate potential therapeutic interventions of brain IR injury [22,23].

## 2. Results

### 2.1. Alleviation of IR-Induced Locomotor Hyperactivity by Porphyran

**Figure 1:**
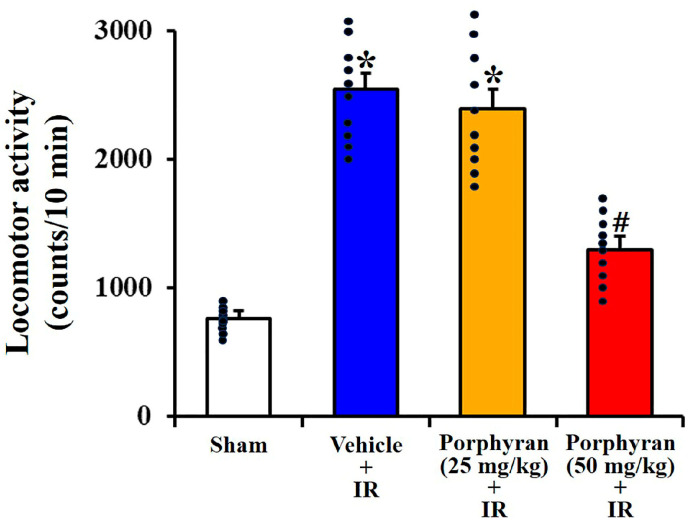
The mean number of photocell beam interruptions per 10 min used to assess SLA in the sham, vehicle–IR, 25 mg/kg, and 50 mg/kg porphyran–IR groups on day 1 after IR. Note that locomotor activity in the 50 mg/kg porphyran–IR group is significantly low in comparison with that in the vehicle–IR group. The error bars represent mean ± SEM (*n* = 10, respectively; * *p* < 0.05 vs. sham group, ^#^ *p* < 0.05 vs. vehicle–IR group).

Spontaneous locomotor activity (SLA), which is a useful parameter reflecting the physical status of experimental animals, was assessed to evaluate the effects of porphyran on IR-induced locomotor hyperactivity at 1 day after IR (Figure 1). Spontaneous locomotor activity was significantly increased (357% of the sham group, as locomotor hyperactivity) in the vehicle–IR group in comparison with the sham group, indicating that locomotor hyperactivity was induced by IR. Locomotor hyperactivity in the 25 mg/kg porphyran–IR group was not different from that evaluated in the vehicle–IR group, whereas, in the 50 mg/kg porphyran–IR group, locomotor hyperactivity was significantly decreased to 48% compared to the vehicle–IR group.

### 2.2. Reduction in IR-Induced Pyramidal Cell Death by Porphyran

#### 2.2.1. Cresyl Violet (CV)-Stained Cells

**Figure 2:**
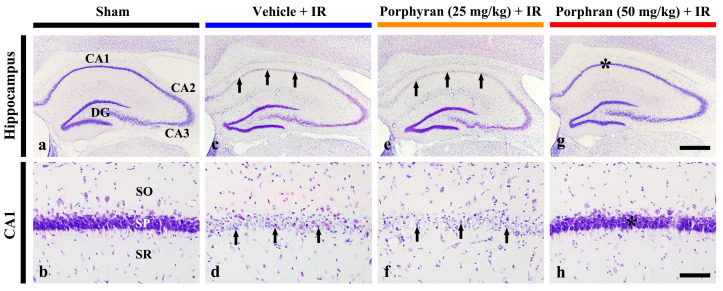
Representative images of CV staining in the hippocampus (upper panels) and its CA1 subregion (lower panels) of the sham (**a**,**b**), vehicle–IR (**c**,**d**), 25 mg/kg (**e**,**f**), and 50 mg/kg porphyran–IR (**g**,**h**) groups at 4 days after IR. Strong CV stainability is observed in the stratum pyramidale (SP, asterisks) consisting of pyramidal cells in the CA1 subregion of the 50 mg/kg porphyran–IR group in comparison with that in the vehicle–IR and 25 mg/kg porphyran–IR groups, showing damaged pyramidal cells (arrows). CA, cornu ammonis; DG, dentate gyrus; SO, stratum oriens; SR, stratum radiatum. Scale bar = 400 µm (**a**,**c**,**e**,**g**) and 50 µm (**b**,**d**,**f**,**h**).

To assess histopathological changes in the hippocampus at 4 days after IR, we conducted CV staining, a technique for staining Nissl bodies (rough endoplasmic reticulum and free ribosomes) in the brain and spinal cord (Figure 2). In the sham group, CV-stained (CV^+^) cells, as intact cells, were distinguished in all subregions of the hippocampus (Figure 2a,b). In the vehicle–IR group, very faint CV stainability was observed in the stratum pyramidale consisting of pyramidal cells (principal neurons) in the CA1 subregion (Figure 2c), and the CV^+^ CA1 pyramidal neurons were shrunken (Figure 2d). This finding indicates that IR caused damage or death of the CA1 pyramidal cells. In the 25 mg/kg porphyran–IR group, CV stainability and morphology in the CA1 pyramidal neurons were not different from those shown in the vehicle–IR group (Figure 2e,f). In the 50 mg/kg porphyran–IR group, however, strong CV stainability was observed in the CA1 pyramidal neurons in comparison with that found in the vehicle–IR group (Figure 2g,h). This finding indicates that the CA1 pyramidal neurons in the 50 mg/kg porphyran–IR group were protected from IR injury.

#### 2.2.2. Neuronal Nuclei (NeuN)^+^ Cells

**Figure 3:**
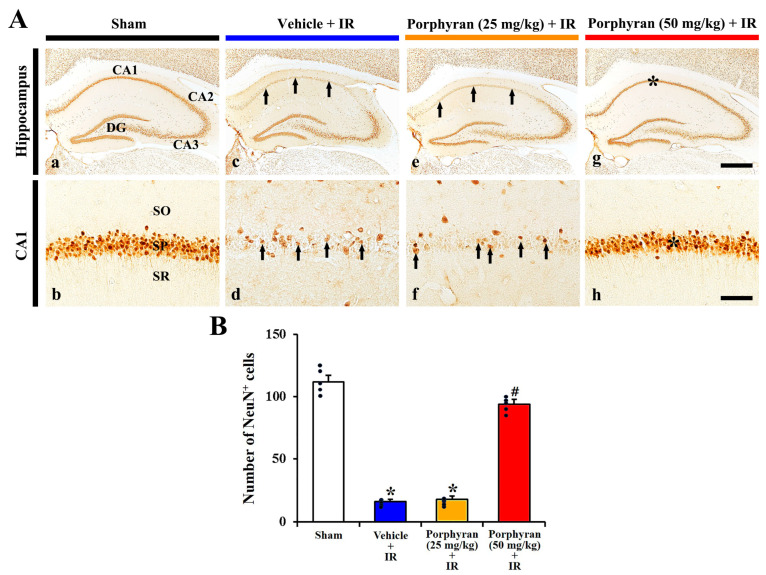
(**A**) Representative images of NeuN immunohistochemistry in the hippocampus (upper panels) and its CA1 subregion (lower panels) of the sham (**a**,**b**), vehicle–IR (**c**,**d**), 25 mg/kg (**e**,**f**), and 50 mg/kg porphyran-IR (**g**,**h**) groups at 4 days after IR. Numerous NeuN^+^ pyramidal cells are shown in the stratum pyramidale (SP, asterisks) of the CA1 subregion in the 50 mg/kg porphyran–IR group as compared with the vehicle–IR and 25 mg/kg porphyran–IR groups, showing few pyramidal cells (arrows). CA, cornu ammonis; DG, dentate gyrus; SO, stratum oriens; SR, stratum radiatum. Scale bar = 400 µm (**a**,**c**,**e**,**g**) and 50 µm (**b**,**d**,**f**,**h**). (**B**) Quantification of NeuN^+^ pyramidal cells in a 250 × 250 μm square of the CA1 subregion. The error bars represent mean ± SEM (*n* = 5, respectively; * *p* < 0.05 vs. sham group, ^#^ *p* < 0.05 vs. vehicle–IR group).

To assess neuronal survival in the CA1 subregion at 4 days after IR, we performed immunohistochemistry for NeuN, which is found in the nuclei and perinuclear cytoplasm of most neurons (Figure 3). NeuN immunoreactivity, in the sham group, was detected in intact pyramidal neurons (principal neurons) consisting of the stratum pyramidale (Figure 3A(a,b)). In the vehicle–IR group, NeuN^+^ pyramidal neurons were rarely detected (16 ± 4.5 cells/250 μm^2^) in the CA1 stratum pyramidale (Figure 3A(c,d),B). In the 25 mg/kg porphyran–IR group, the number of NeuN^+^ CA1 pyramidal cells was not different from that evaluated in the vehicle–IR group (Figure 3A(e,f),B). However, in the 50 mg/kg porphyran–IR group, there were substantial numbers of NeuN^+^ CA1 pyramidal neurons, 94 ± 6.3 cells/250 μm^2^ (Figure 3A(g,h),B), indicating that porphyran treatment protected CA1 pyramidal neurons against IR injury.

### 2.3. Inhibition of IR-Induced Microgliosis by Porphyran

**Figure 4:**
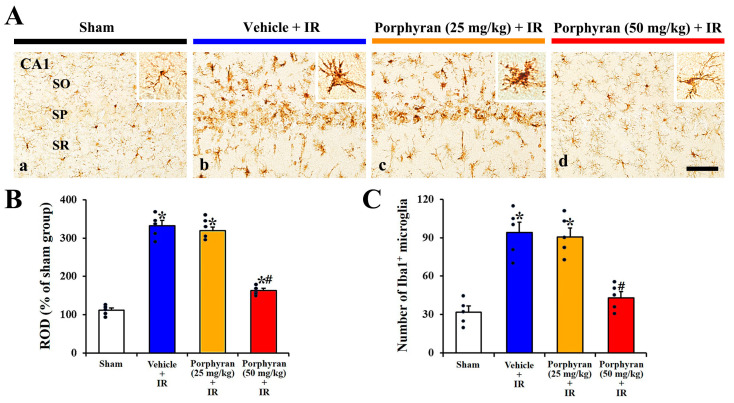
(**A**) Representative images of Iba1 immunohistochemistry in the CA1 subregion of the sham (**a**), vehicle–IR (**b**), 25 mg/kg, and 50 mg/kg porphyran–IR (**c**,**d**) groups on day 4 after IR. In the sham group, normal Iba1^+^ microglia are presented with a higher magnification (in a smaller panel). In the vehicle and 25 mg/kg porphyran–IR groups, microgliosis (activation and proliferation of Iba1+ microglia) is apparent, but the microgliosis is distinctly attenuated in the 50 mg/kg porphyran–IR group. SO, stratum oriens; SR, stratum radiatum. Scale bar = 50 µm. (**B**,**C**) ROD of Iba1 immunoreactivity (**B**) and the number of Iba1+ microglia (**C**). The error bars represent mean ± SEM (*n* = 5, respectively; * *p* < 0.05 vs. sham group, ^#^ *p* < 0.05 vs. vehicle–IR group).

To investigate changes in microglioses (activation and proliferation of microglia) in the CA1 subregion at 4 days after IR, immunohistochemistry for ionized calcium binding adapter protein 1 (Iba1, a microglial marker) was conducted (Figure 4A). In the sham group, Iba1^+^ microglia were distributed throughout the CA1 subregion, and they possessed small cell bodies with long and thin ramified processes, indicating a resting state (Figure 4A(a)). In the vehicle–IR group, Iba1^+^ microglia displayed enlarged cell bodies and thickened processes, indicating that microglia were activated after IR (Figure 4A(b)). Notably, many activated Iba1^+^ microglia were gathered in the stratum pyramidale where IR-induced neuronal death occurred (Figure 4A(b)). In the 25 mg/kg porphyran–IR group, the morphology and distribution of Iba1^+^ microglia were similar to that in the vehicle–IR group (Figure 4A(c)), indicating that treatment with 25 mg/kg porphyran failed to achieve neuroprotection against IR injury. However, in the 50 mg/kg porphyran–IR group, microgliosis induced by IR was distinctly alleviated (Figure 4A(d)).

To clarify the changes in microgliosis, the relative optical density (ROD) of Iba1 immunoreactivity and numbers of Iba1^+^ microglia were evaluated (Figure 4B,C). In the vehicle–IR group, the ROD (approximately 343%) and the number (95 ± 8.2 cells) were considerably increased in comparison with those evaluated in the sham group. In the 25 mg/kg porphyran–IR group, the ROD and the number did not differ significantly from those of the vehicle–IR group. However, in the 50 mg/kg porphyran–IR group, the ROD (approximately 160%) and the number (42 ± 5.1 cells) were significantly lower than those of the vehicle–IR group.

### 2.4. Attenuation of IR-Induced NLRP3 Inflammasome-Mediated Neuroinflammation by Porphyran

**Figure 5:**
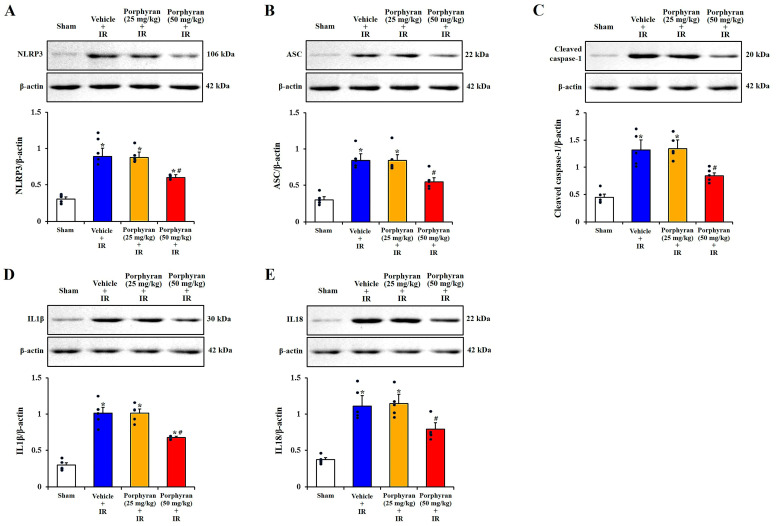
(**A**–**E**) Representative Western blot images and quantitative analyses of NLRP3 (**A**), ASC (**B**), cleaved caspase-1 (**C**), IL1β (**D**), and IL18 (**E**) in the CA1 subregion extracted from the sham, vehicle–IR, 25 mg/kg, and 50 mg/kg porphyran–IR groups on day 4 after IR. Note that original images of Western blot bands are included in the Supplementary Materials Figures S1–S5. Protein expression levels of NLRP3, ASC, cleaved caspase-1, IL1β, and IL18 in the 50 mg/kg porphyran–IR group are significantly lower than those evaluated in the vehicle–IR group. The error bars represent mean ± SEM (*n* = 5, respectively; * *p* < 0.05 vs. sham group, ^#^ *p* < 0.05 vs. vehicle–IR group).

To examine changes in neuroinflammation mediated by the NLRP3 inflammasome (a multiprotein complex involved in the inflammatory response) in the CA1 subregion at 4 days after IR, Western blot analysis to detect the protein expressions of NLRP3 inflammasome components (NLRP3, apoptosis-associated speck-like protein containing a caspase recruitment domain (ASC) and cleaved caspase-1, as well as pro-inflammatory cytokines (IL1β, and IL18)) was performed (Figure 5A–E). In the vehicle–IR group, the protein levels of NLRP3, ASC, cleaved caspase-1, IL1β, and IL18 were significantly elevated (3.8, 2.6, 3.1, 3.2, and 3.0 times, respectively) in comparison with those of the sham group. The protein levels, in the 25 mg/kg porphyran–IR group, did not differ from those estimated in the vehicle–IR group. However, in the 50 mg/kg porphyran–IR group, the protein levels of NLRP3, ASC, cleaved caspase-1, IL1β, and IL18 were considerably lower (approximately 55%, 36%, 50%, 44%, and 32%, respectively) in comparison with those evaluated in the vehicle–IR group.

## 3. Discussion

Growing evidence shows that pretreatment with polysaccharides derived from marine algae exhibits robust neuroprotective effects against cerebral IR injury [24,25]. However, the potential neuroprotective effects of porphyran pretreatment against cerebral IR injury are not fully known. Therefore, we conducted the first evaluation of whether pretreated porphyran can provide neuroprotection in gerbils subjected to brain IR injury, which elicits a considerable pyramidal cell loss in the hippocampal CA1 subregion on day 4 after forebrain IR [7,8].

When the SLA test is appropriately designed and analyzed, it serves as a valuable tool for detecting locomotor hyperactivity and understanding the underlying factors contributing to abnormal motor behavior in research subjects. It is well established that post-ischemic locomotor hyperactivity is a typical behavioral change observed in the early stages following brain IR [26]. In the hippocampal CA1 subregion following IR, locomotor hyperactivity has been used as an important predictor of pyramidal cell death [27,28]. In this study, we examined the effect of porphyran on IR-induced locomotor hyperactivity at 1 day after IR using an SLA test and found that IR-induced locomotor hyperactivity was significantly alleviated by pretreatment with 50 mg/kg of porphyran, but not 25 mg/kg of porphyran.

Based on the finding of alleviation of the locomotor hyperactivity by porphyran, we evaluated whether pretreatment with porphyran protected the CA1 pyramidal cells from IR injury at 4 days after IR using CV staining and NeuN immunohistochemistry. The results revealed that pretreatment with 50 mg/kg of porphyran, not 25 mg/kg of porphyran, significantly protected the CA1 pyramidal cells from IR injury. These findings indicate that porphyran possesses neuroprotective activity against cerebral IR injury. It demonstrated that pretreatment with fucoidan, a sulfated polysaccharide of brown algae, protected the liver from hepatic IR injury in rats [29] and provided protection against transient forebrain ischemic injury in gerbils [14]. Taken together, we suggest that a sulfated polysaccharide derived from algae like porphyran and fucoidan possesses protective activity against IR injury in brain and livers.

Microglia (microglial cells) are the resident innate immune cells located in the central nervous system (brain and spinal cord) and play vital roles in regulating key pathways in neuroinflammation [30,31]. Accumulating evidence indicate that microgliosis, the activation and proliferation of microglia, in ischemic brain regions represents a common pathological feature of IR injury [32,33]. Activated microglia release excessive pro-inflammatory cytokines, which are involved in neuronal death caused by cerebral IR injury [34,35]. Based on the aforementioned studies, it is suggested that suppressing microglia activation and proliferation may be a key target for neuroprotective strategies against cerebral IR injury [36,37]. In this connection, we recently demonstrated that pretreatment with fucoidan and laminarin, polysaccharides present in brown algae, significantly attenuated IR-induced microglia activation in the hippocampal CA1 subregion of the gerbil, contributing to neuroprotection against brain IR injury [14,15]. Similarly, our current study showed that pretreatment with 50 mg/kg porphyran considerably inhibited the activation and proliferation of microglia induced by IR in the gerbil hippocampal CA1 subregion. Thus, together with these findings, our current results indicate the possibility that the attenuation of IR-induced microglia proliferation and activation following porphyran treatment contributes to protecting hippocampal CA1 pyramidal cells from cerebral IR injury.

Relevant players in neuroinflammation, such as microglia/monocytes, show a powerful activation of the NLRP3 inflammasome [38]. In particular, NLRP3 inflammasome is predominantly expressed in microglia in the context of ischemic brain injury and plays a decisive role in this process [39,40]. NLRP3 inflammasome, as a multi-protein complex, can be activated by various damage- or pathogen-related molecular patterns, and the improper activation of NLRP3 inflammasome can lead to autoinflammatory, autoimmune, or metabolic disorders [41]. NLRP3 is an intracellular sensor detecting endogenous danger signals, broad microbial motifs, and environmental irritants, resulting in the formation and activation of the NLRP3 inflammasome, which comprises sensor NLRP3, adaptor ASC, and effector caspase-1 [42,43]. Upon activation, the inflammasome induces the activation and cleavage of caspase-1, ultimately resulting in the excessive production of pro-inflammatory cytokines IL1β and IL18 during the pathological process of cerebral IR injury [40,44]. In this context, Wang et al. (2023) showed that, in mice, NLRP3 knockdown with small interfering RNA significantly reduced neurological deficits and brain infarction induced by transient focal cerebral ischemia [45]. Additionally, some studies revealed that pharmacological inhibition of NLRP3 inflammasome-mediated inflammatory response displayed strong neuroprotective effects against transient focal cerebral ischemia injury in mice and rats [40,46,47]. In accordance with these studies, our current study showed that brain IR in the gerbil significantly increased protein expression levels of NLRP3-inflammasome components (NLRP3, ASC, and cleaved caspase-1) and pro-inflammatory cytokines (IL1β and IL18) in the CA1 subregion with IR injury, but the increased protein expression levels were significantly attenuated by pretreatment with 50 mg/kg porphyran. Taken together, we suggest that porphyran can significantly attenuate NLRP3 inflammasome-mediated neuroinflammation in cerebral IR injury.

## 4. Materials and Methods

### 4.1. Ethical Statement and Experimental Animals

All experimental procedures were permitted (Permit Number, KW-2000113-1) under the security guidelines of our Institutional Animal Care and Use Committee (Kangwon National University). Adult male gerbils (6.5 months old), weighing 70 ± 5 g, were used, and these had been bred at our Experimental Animal Center. They were acclimated for 7 days prior to the start of the experiment under controlled environmental conditions (temperature, 24 ± 2 °C of temperature; 56 ± 5% of humidity; 08:00 to 20:00 h of lighting) with unrestricted access to food and water. In particular, all efforts were made to minimize any distress or pain experienced by the animals over the course of the experiment.

Forty gerbils were randomly allocated into four different groups (*n* = 10/group; 5 for histology, 5 for western blotting): (1) vehicle-treated and sham-operated group (sham group); (2) vehicle-treated and IR-operated group (vehicle–IR group); (3) 25 mg/kg porphyran-treated and IR-operated group (25 mg/kg porphyran–IR group); (4) 50 mg/kg porphyran–IR group.

### 4.2. Administration of Porphyran

**Figure 6:**
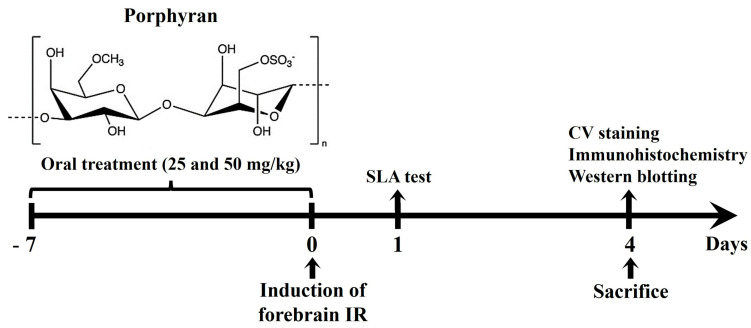
The chemical structure of porphyran and experiment schedule. Gerbils are subjected to IR injury, and porphyran (25 and 50 mg/kg) is orally administered once daily for 7 days before IR induction. SLA is assessed on day 1 after IR injury. On day 4 after IR injury, the gerbils are sacrificed, and their brain samples are collected for histological and biochemical analyses. IR, ischemia and reperfusion in the forebrain; CV, cresyl violet.

The chemical composition of porphyran and the experiment schedule are illustrated in Figure 6. The doses of porphyran (Biosynth International, Inc., San Diego, CA, USA) were selected based on previous papers that showed that the administration of 25 and 50 mg/kg of porphyran prevented dopaminergic neuronal death in a mouse model of Parkinson’s disease, which was induced by 1-methyl-4-phenyl-1,2,3,6-tetrahydropyridine [20]. The gerbils of the 25 and 50 mg/kg porphyran–IR groups were orally administered 0.3 mL of saline containing the corresponding dose of porphyran using a feeding needle once a day for one week before the induction of forebrain IR. In the sham and vehicle–IR groups, the gerbils were treated with 0.3 mL of saline (vehicle) without porphyran according to the same schedule.

### 4.3. Induction of IR Injury

IR injury was induced in the forebrains of the gerbils using a previously described method [16]. Shortly, the animals were appropriately anesthetized using 2.5% isoflurane obtained from Pharmaceutical Corporation (Seoul, Republic of Korea). An incision was made in the ventral surface of the neck and isolated two (right and left) common carotid arteries, which supply blood to the brain. The arteries were occluded with aneurysm clips (Fine Science Tools, Foster City, CA, USA) for 5 min. Blood flow interruption was confirmed by observing blood flow within the central retinal arteries (the end branches of the common carotid arteries) under a HEINE K180 ophthalmoscope (Heine Optotechnik, Herrsching, Germany). After the confirmation of a perfect stop of blood flow, the clips were taken off to restore blood flow. The gerbils’ body temperatures were maintained at 37 ± 0.5 °C during the surgery using a thermostat blanket and an infrared radiation lamp. To avoid hypothermia, the gerbils were monitored for an additional 4 h after the surgery. The gerbils used for the sham group underwent the same surgical procedure, without the common carotid artery occlusion.

### 4.4. SLA Test

The test of SLA was assessed on day 1 after forebrain IR surgery using a previously published procedure [48]. Briefly, each gerbil was placed in a 25 × 20 × 12 cm Plexiglas cage equipped with infrared-sensitive photocells. The number of beam breaks per min was measured by the photobeam activity system (San Diego Instruments, San Diego, CA, USA), which is used as a measure of SLA. This apparatus was installed in a dark and sound-alleviated room. The movement of each gerbil was evaluated through the interruption of an array of 32 infrared beams generated by photocells. In this experiment, before the SLA test, each gerbil was kept in a Plexiglas cage for 5 min to be habituated. Thereafter, SLA was recorded for 10 min and expressed as total photobeam counts for each 10 min period.

### 4.5. Tissue Preparation for Microscopic Observations

In accordance with a previously described procedure [49], all gerbils were deeply anesthetized with 1.5 g/kg of urethane (an intraperitoneal injection; Sigma-Aldrich, St. Louis, MO, USA). Immediately, they underwent intracardiac perfusion with saline and were fixed with 4% paraformaldehyde. Their brains were then harvested, post-fixed in 4% paraformaldehyde overnight, and cryoprotected in a 30% sucrose solution. The brain tissues containing the hippocampi were coronally cut into 30 μm thicknesses using a cryostat (Leica, Wetzlar, Germany) and placed into storage solution at 4 °C. The sections used in this experiment were chosen at −1.4 mm to −2.2 mm levels antero-posteriorly to the bregma, based on the gerbil brain atlas [50].

### 4.6. CV Staining

Histochemical staining with CV was conducted in the hippocampus on day 4 after IR. In brief, as described previously [49], the tissue sections containing the hippocampi were attached to slide glasses and hydrated through a series of ethanol solutions. They were then stained with 0.1% CV (Sigma-Aldrich) for 18–22 min at room temperature, followed by rinsing in distilled water. Next, decolorization was performed through a series of ethanol solutions, cleared with xylene, and covered with Canada balsam (Sigma-Aldrich) and coverslips. The CV^+^ sections were inspected with BX53 light microscopy (Olympus, Tokyo, Japan).

### 4.7. Immunohistochemistry

To evaluate neuronal survival and microgliosis in the ischemic CA1 subregion, immunohistochemical staining was conducted at 4 days after IR. With a previously published method [14], the prepared brain sections were blocked with 10% normal chicken serum (Vector, Burlingame, CA, USA) diluted in 0.01 M phosphate-buffered saline (PBS) for 25 min at room temperature. The sections were then incubated in the following primary antibodies: mouse anti-NeuN (diluted 1:1000, Chemicon, Temecula, CA, USA) or rabbit anti-Iba1 (diluted 1:800, Wako, Osaka, Japan). They were washed before donkey anti-mouse or rabbit IgG (diluted 1:250, Vector) was reacted as the secondary antibody. Signals were amplified using an avidin–biotin complex (diluted 1:250, Vector). After the sections were allowed to react with 0.02% 3,3′-diaminobenzidine tetrahydrochloride (Sigma-Aldrich), they were coverslipped with Canada balsam (Sigma-Aldrich).

The quantification of NeuN^+^ and Iba1^+^ cell was assessed using a previously published protocol [32]. In brief, three sections per gerbil were collected at 100 μm intervals for the analysis of the NeuN^+^ and Iba1^+^ cells. Photomicrographs of each section, including the stratum oriens, stratum pyramidale, and stratum radiatum, were taken at ×200 magnification using BX53 light microscopy (Olympus), equipped with a DP72 digital camera (Olympus). The number of NeuN^+^ and Iba1^+^ cells per square was counted within 250 × 250 μm and averaged using an image capture software 1.18 version (cellSens Standard, Olympus).

To quantitatively analyze Iba1 immunoreactivity, three sections per gerbil were selected and analyzed as previously described [14]. In short, photomicrographs of Iba1^+^ structures (cell bodies and their processes) were captured using the aforementioned protocol. The images were converted into a resolution of 512 × 512 pixels, with each pixel representing 256 Gy levels. Iba1 immunoreactivity (intensity of Iba1) was measured by the relative optical density (ROD), calculated using a formula of ROD = log_10_ (256/mean grayscale level). The ROD for background staining was assessed in the unlabeled part of the CA1 subregions with Photoshop CC 2018 software (Adobe Systems Inc., San Jose, CA, USA). The value was then subtracted to adjust for nonspecific staining, utilizing ImageJ software 1.52 version (NIH). The data are presented as percentages of the values from the sham group, which were set to 100%.

### 4.8. Western Blotting

To examine NLRP3 inflammasome-mediated neuroinflammation in the hippocampal CA1 subregion on day 4 after forebrain IR, we conducted Western blotting according to the method described previously [51]. Briefly, after sacrificing the gerbils under deep anesthesia with 1.5 g/kg of urethane, the hippocampal CA1 subregions were harvested. The total protein was extracted from the tissues using a radioimmunoprecipitation assay lysis buffer obtained from Thermo Fisher Scientific (Waltham, MA, USA). Protein samples, each weighing 30 μg, were equally divided and subjected to separation using sodium dodecyl sulfate-polyacrylamide gel electrophoresis, followed by transfer onto nitrocellulose membranes. To minimize non-specific antibody binding, the membranes were blocked in 5% skim dry milk or bovine serum albumin in Tween20/PBS. Subsequently, they were incubated overnight at 4 °C with the following protein-specific antibodies: rabbit anti-NLRP3 (diluted 1:1000, Abcam, Cambridge, UK), rabbit anti-ASC (diluted 1:1000, Abcam), rabbit anti-cleaved caspase-1 (diluted 1:500, Cell Signaling Technology, Danvers, MA, USA), rabbit anti-IL1β (diluted 1:1000, Abcam), rabbit anti-IL18 (diluted 1:500, Abcam), and rabbit anti-β-actin (diluted 1:5000, Sigma-Aldrich). After incubation with horseradish peroxidase-conjugated goat anti-rabbit secondary antibody (diluted 1:250; Vector), the proteins were visualized by following the manufacturer’s instructions for enhanced chemiluminescence (Pierce Chemical, Dallas, TX, USA). The immunoreactive bands were analyzed densitometrically using NIH ImageJ 1.59 software.

### 4.9. Statistical Analysis

All data are presented as means ± standard error of the mean (SEM). One-way ANOVA and Tukey’s multiple comparisons test in GraphPad Prism 5.0 (GraphPad Software Inc., San Diego, CA, USA) were used to determine statistical significance for between-group differences. A *p* value < 0.05 was considered statistically significant.

## 5. Conclusions

Porphyran is a sulfated polysaccharide contained in various species of red algae and it exhibits various bioactivities, including anti-inflammatory effects. In this study, locomotor hyperactivity and pyramidal cell loss in the hippocampal CA1 subregion were found on day 1 and 4, respectively, after brain IR in gerbils. Porphyran (25 and 50 mg/kg) was orally administered daily for one week prior to IR. Pretreatment with 50 mg/kg porphyran significantly attenuated locomotor hyperactivity and protected CA1 pyramidal cells from IR injury in gerbils, and the pretreatment significantly suppressed IR-induced microgliosis in the ischemic CA1 subregion through inhibiting the overexpressions of NLRP3 inflammasome components (NLRP3, ASC, and cleaved caspase-1) and pro-inflammatory cytokines (IL1β and 18) induced by IR. These results indicate that neuroprotection against brain IR injury can be displayed by porphyran pretreatment through strongly correlating with the attenuation of NLRP3 inflammasome-mediated neuroinflammation, suggesting that porphyran has the potential to serve as a preventive agent against cerebral IR injury.

## Data Availability

The data presented in this study are available on request from the corresponding author.

## Supplementary Materials

The following supporting information can be downloaded at https://www.mdpi.com/article/10.3390/md22040170/s1, Figures S1–S5: Original images of Western blot data in Figure 5.
