# Supplementary material for: Porphyran Attenuates Neuronal Loss in the Hippocampal CA1 Subregion Induced by Ischemia and Reperfusion in Gerbils by Inhibiting NLRP3 Inflammasome-Mediated Neuroinflammation
Source: Mar Drugs. 2024 Apr 11;22(4):170. doi: 10.3390/md22040170 (PMC11050983; doi:10.3390/md22040170)
Supplement: Supplementary file 1 [file marinedrugs-22-00170-s001.zip › marinedrugs-2946895-supplementary.pdf]

Porphyran Attenuates Neuronal Loss in the Hippocampal CA1 Subregion Induced by Ischemia and Reperfusion in Gerbils by Inhibiting NLRP3 Inflammasome-Mediated Neuroinflammation

Dae Won Kim 1,†, Tae-Kyeong Lee 2,†, Ji Hyeon Ahn 3, Se-Ran Yang 4, Myoung Cheol Shin 5, Jun Hwi Cho 5, Moo-Ho Won 5, Il Jun Kang 2,* and Joon Ha Park 6,*

1 Department of Biochemistry and Molecular Biology, Research Institute of Oral Sciences, College of Dentistry, Gangneung-Wonju National University, Gangneung 25457, Republic of Korea; kimdw@gwnu.ac.kr

2 Department of Food Science and Nutrition, Hallym University, Chuncheon 24252, Republic of Korea; tk_lee@hallym.ac.kr

3 Department of Physical Therapy, College of Health Science, Youngsan University, Yangsan 50510, Republic of Korea; jh-ahn@ysu.ac.kr

4 Department of Cardiovascular Surgery, School of Medicine, Kangwon National University, Chuncheon 24341, Republic of Korea; seran@kangwon.ac.kr

5 Department of Emergency Medicine, Kangwon National University Hospital, School of Medicine, Kangwon National University, Chuncheon 24289, Republic of Korea; dr10126@naver.com (M.C.S.); cjhemd@kangwon.ac.kr (J.H.C.); mhwon@kangwon.ac.kr (M.-H.W.)

6 Department of Anatomy, College of Korean Medicine, Dongguk University, 123 Dongdae-ro, Gyeongju 38066, Republic Korea

* Correspondence: ijkang@hallym.ac.kr (I.J.K.); jh-park@dongguk.ac.kr (J.H.P.); Tel: +82-33-248-2135 (I.J.K.); +82-54-770-2369 (J.H.P.); Fax: +82-33-255-4787 (I.J.K.)

† These authors contributed equally to this work.

Figure S1

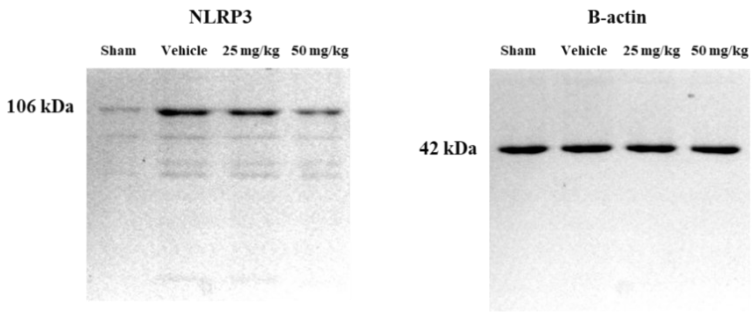

Figure S2

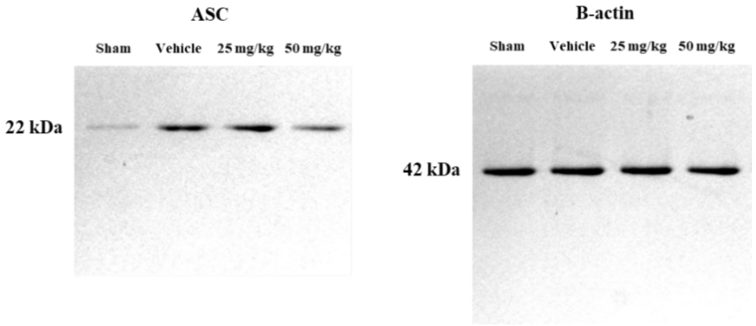

Figure S3

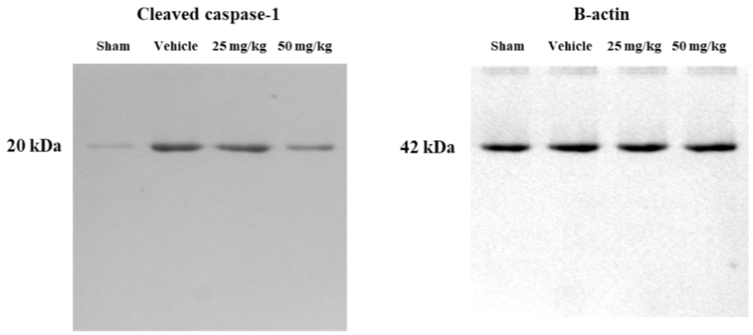

Figure S4

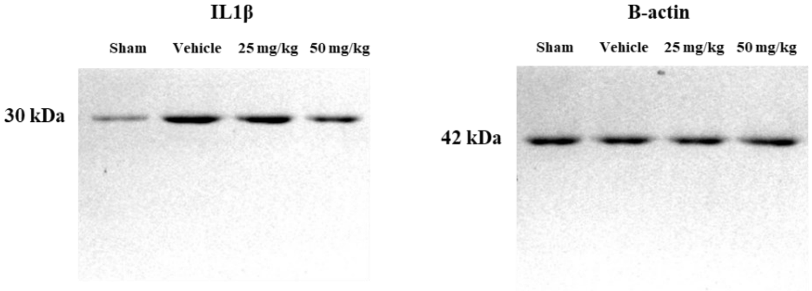

Figure S5

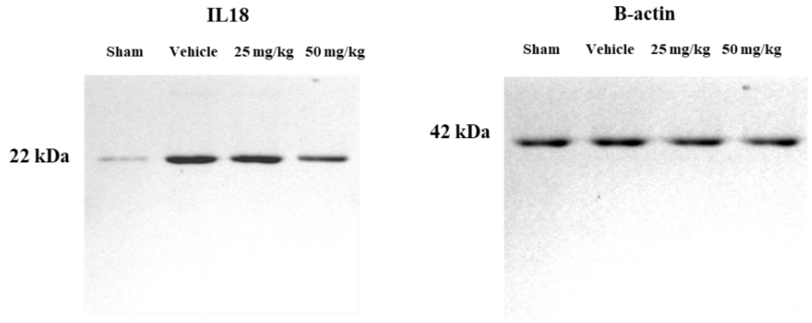

Supplementary Figures S1-S5. Original images of Western blot data in Figure 5
